# E-cadherin re-expression shows *in vivo* evidence for mesenchymal to epithelial transition in clonal metastatic breast tumor cells

**DOI:** 10.18632/oncotarget.9715

**Published:** 2016-05-30

**Authors:** Katie Palen, James Weber, Michael B. Dwinell, Bryon D. Johnson, Ramani Ramchandran, Jill A. Gershan

**Affiliations:** ^1^ Department of Pediatrics at the Medical College of Wisconsin, Milwaukee, Wisconsin 53226, USA; ^2^ Department of Microbiology and Molecular Genetics at the Medical College of Wisconsin, Milwaukee, Wisconsin 53226, USA; ^3^ Department of Obstetrics and Gynecology at the Medical College of Wisconsin, Milwaukee, Wisconsin 53226, USA

**Keywords:** breast cancer, EMT, MET, metastasis, E-cadherin

## Abstract

Substantial experimental evidence has shown that dedifferentiation from an epithelial state to a mesenchymal-like state (EMT) drives tumor cell metastasis. This transition facilitates tumor cells to acquire motility and invasive features. Intriguingly, tumor cells at the metastatic site are primarily epithelial, and it is believed that they differentiate back to an epithelial state by a process called mesenchymal to epithelial transition (MET). However, there is little *in vivo* evidence to support the MET process. To investigate EMT and MET *in vivo*, we generated two epithelial (E) and two mesenchymal (M) primary clonal cell lines from a spontaneous mouse mammary tumor (Tg MMTV/neu). These cells were labeled with reporters (GFP and luciferase), and tracked *in vivo* during primary tumor growth and subsequent secondary metastasis. Once E cells were implanted into the mammary fat pad, E-cadherin expression progressively decreased and continued to decrease as the primary tumor enlarged over time. A greater percentage of E tumor cells expressed E-cadherin at the secondary metastatic site as compared to the corresponding primary tumor site. Collectively, these data provide direct *in vivo* evidence that epithelial tumor cells have metastatic potential, undergo EMT at the primary tumor site, and MET at the metastatic site.

## INTRODUCTION

The metastatic process of tumor cell dissemination and colonization at a distant organ site is a critical process in tumor progression, and is poorly understood. Carcinomas are epithelial cell-derived tumors, yet they are composed of a heterogeneous mix of tumor cells that are epithelial and mesenchymal-like. Substantial evidence suggests that phenotypic plasticity allows for dedifferentiation of epithelial tumor cells resulting in expression of mesenchymal lineage markers [1–7]. This process is referred to as an epithelial to mesenchymal transition or EMT. Decades of research suggest a paradigm that EMT is a prerequisite process for carcinoma cell metastasis. EMT is characterized by expression of EMT-inducing transcription factors such as SNAIL1/2, TWIST1/2, ZEB1/2, FOXC2, SOX4, TEAD2, LHX2 and PRRX1; upregulation of fibronectin and vimentin, downregulation of E-cadherin and a change from epithelial cuboidal cell sheets to a morphology that is spindle-shaped and fibroblast-like as reviewed in [8]. The EMT process is associated with tumor cell migration, invasion, metastasis and a poor clinical outcome [9–16]. A long-standing conundrum is that dedifferentiated mesenchymal-like tumor cells originating in the primary tumor are believed to be the cells that metastasize. However, tumor cells at corresponding metastatic sites are not dedifferentiated, but instead, are differentiated toward an epithelial phenotype [17–22]. In fact, metastatic cells can show greater epithelial differentiation (increased E-cadherin expression) as compared to cells in the corresponding primary orthotopic tumor [23, 24]. To explain this observation, it is hypothesized that mesenchymal-like metastatic cells revert back to an epithelial phenotype via a mesenchymal to epithelial transition (MET) when colonizing at the distant site. MET can be induced *in vitro* and there is evidence that MET occurs *in vivo*. The natural bioactive molecule, honokiol, reversed the acquisition of mesenchymal characteristics in TGF-β and TNF-α stimulated normal mammary epithelial MCF10A cells, and induced MET in human kidney carcinoma and gastric cell lines [25–27]. In addition, loss of the PRRX1 transcription factor induced MET in human breast cancer BT-549 cells [16]. *In vivo* data has demonstrated the association of MET with tumor cell colonization and metastasis. In a reversible EMT model, Twist1 expression induced EMT while subsequent repression of Twist1 reversed EMT. This on, and off mechanism in terms of Twist1 expression was required for macrometastasis of murine squamous cell carcinoma [28]. Other examples of preclinical data include: Non-metastatic 4T07 breast tumor cells formed metastases when they expressed MiR-141-200c and E-cadherin [29]. Downregulation of E-cadherin in human TSU-pr1-B2 bladder cancer cells inhibited distant organ colonization [30]. Upregulation of E-cadherin in human prostate cancer PC-3/S cells enhanced tumorigenicity [31]. While these studies support MET as a requirement for tumor cell colonization/metastasis, direct *in vivo* evidence for this process is lacking.

There are technical problems that must be overcome in order to address the knowledge gaps regarding MET and metastasis. Some of these problems include (1) challenges in distinguishing mesenchymal tumor cells from non-tumor mesenchymal stromal cells, (2) the inability to identify partial or transient EMT or MET in primary tumor and metastatic lesions, respectively, and, (3) difficulty tracking tumor cells in the primary growth phase to metastasis *in vivo*. We have attempted to overcome these technical roadblocks by generating a model system that allows for tracking of primary breast cancer tumor cells *in vivo*. We produced clonal tumor cell lines derived from a spontaneous mammary tumor in a female FVB/N-Tg(MMTVneu) mouse. Tumor-derived cells were genetically modified to express luciferase and fluorescent proteins and were tracked *in vivo* during primary orthotopic tumor growth and subsequent metastasis. These cells were extensively molecularly characterized to be tumor-derived and either epithelial or mesenchymal-like. From this model system, our data shows that (1) over time, epithelial tumor cells undergo EMT changes (including loss of E-cadherin expression) during primary tumor growth, (2) the orthotopically implanted primary clonal epithelial tumor cells are metastatic, and (3) E-cadherin is re-expressed in metastatic tumor cells. To our knowledge, these are the first data to show direct evidence of EMT and MET by tracking clonal epithelial tumor cells *in vivo*.

## RESULTS

### The clonal primary tumor cell lines that were generated as a tool to track EMT and MET changes are tumorigenic *in vivo*

**Figure 1:**
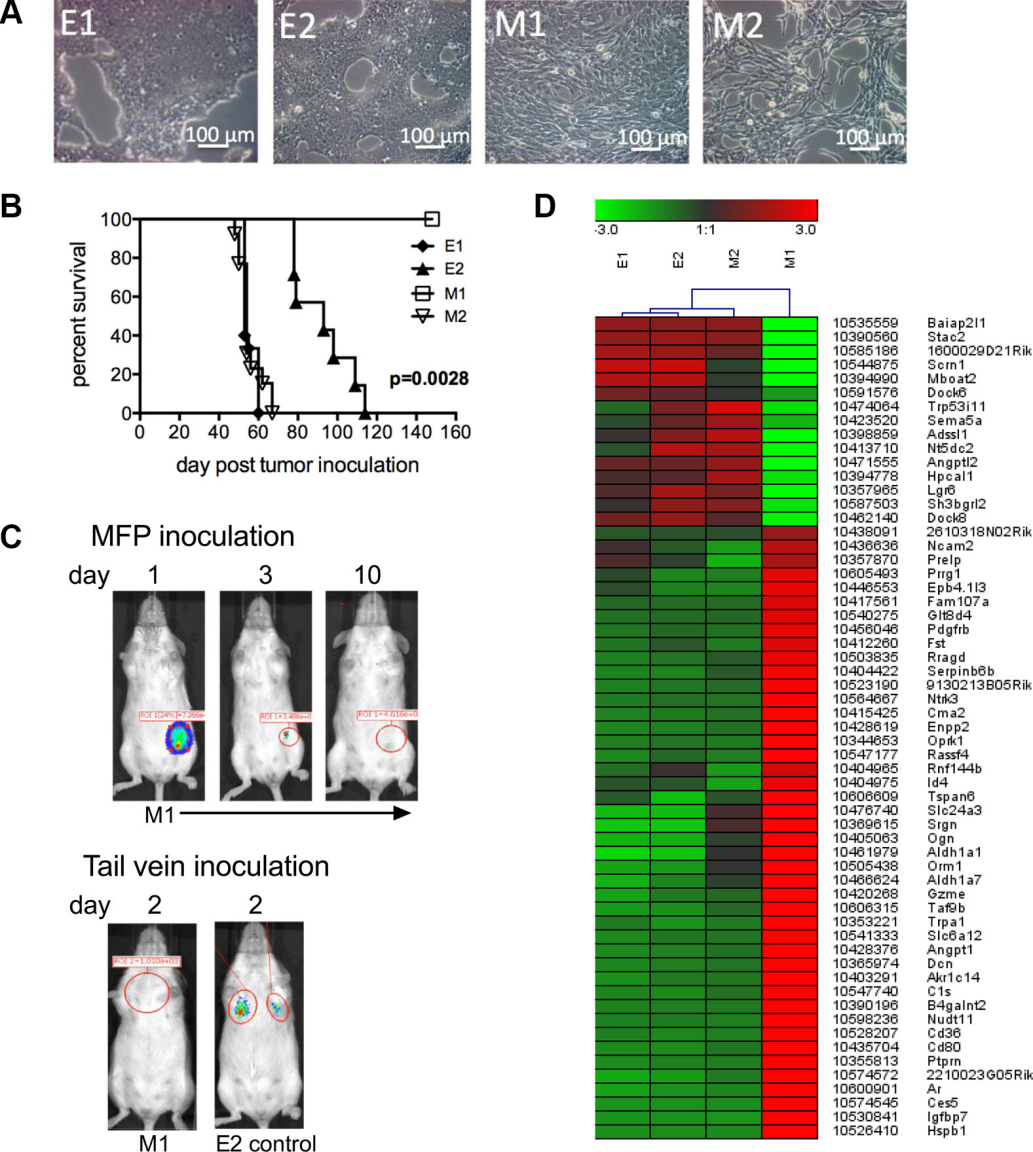
E1, E2 and M2 cells are tumorigenic (**A**) Bright field microscopy of clonal epithelial (E1 and E2) and mesenchymal-like (M1 and M2) cells. (**B**) Survival curves of mice inoculated into the MFP with 5 × 10^4^ cells from each of the E1 (*n* = 15), E2 (*n* = 7), M1 (*n* = 4) and M2 (*n* = 13) cell lines. Data was combined from 2 experiments. Mice were euthanized when tumor size reached 250 mm^2^. Significance was determined using the log-rank test. (**C**) Renilla luciferase bioluminescence in mice inoculated with 2 × 10^6^ M1 cells into the MFP or tail vein. Inoculation of E2 cells into the tail vein was used as a control. Figure C represents data collected from 4 mice. (**D**) Heat map of microarray analysis showing differences in E1, E2, M1 and M2 transcript expression.

To track EMT and MET changes *in vivo*, we generated clonal primary tumor cell lines from a spontaneous breast tumor that arose in a female FVB/N Tg (MMTV/neu) (referred to as Tg/neu) mouse. These mice express the rat ErbB2 (neu) transgene under the control of the mammary-specific mouse mammary tumor virus promoter (MMTV), and develop spontaneous mammary tumors. Cells were sorted from single cell suspensions by flow cytometry based on neu and CD24 expression to separate epithelial and mesenchymal-like cells from the tumor mass. Epithelial tumor cells were enriched in the neu^hi^CD24^hi^ population and mesenchymal-like cells were enriched in the neu^low^CD24^low^ cell population [7]. Two clonal epithelial cell lines (E1 and E2) and 2 clonal mesenchymal-like cell lines (M1 and M2) were obtained by limiting dilution cell culture. Figure 1A shows a morphologic representation of the cell lines. To investigate whether clonal tumor cells were tumorigenic *in vivo*, we orthotopically implanted 50,000 cells from each cell line into the “D” mammary fat pad (MFP). When tumors reached 250 mm^2^ in size, mice were considered moribund and euthanized. Figure 1B shows the Kaplan-Meier survival curves of the various mouse groups. Cells from the epithelial cell lines (E1 and E2) and the M2 mesenchymal-like cell line formed tumors in the MFP, and the mice eventually died from tumor burden. Interestingly, the M1 cell line did not form orthotopic tumor. To facilitate additional analyses, we transduced E cells with GFP and firefly luciferase, and M cells with mCherry and Renilla luciferase. The dual reporter system served purposes of cell sorting (GFP/mCherry), and *in vivo* live cell tracking (firefly/Renilla luciferase) at primary orthotopic and metastatic sites.

We further investigated whether M1 cells colonize to form tumor. We injected 2 × 10^6^ M1 cells transduced to express Renilla luciferase into the MFP, and imaged mice over time. Figure 1C, (panel 1) shows luciferase signal emitted from M1 cells one day after tumor cell inoculation, but the biophotonic signal disappeared within 10 days. We next injected 2 × 10^6^ M1 or 2 × 10^6^ E2 cells (as a control) via tail vein, and two days later we observed no biophotonic signal in the lungs of mice injected with the M1 cells, however signal was present in the lungs of mice injected with E2 cells (Figure 1C, panel 2). We next performed microarray comparisons between the cell lines. The transcripts selected for the heat map had at least a 10-fold difference in expression in the M1 and M2 cells (Figure 1D). These transcripts were also reciprocally regulated in cells that formed tumor (E1, E2 and M2) and cells that did not form tumor (M1). Taken together, these data suggest that E1, E2 and M2 cells share similarity in transcript expression that may contribute to their tumorigenic potential.

### E and M cells are clonally derived from primary mammary tumor

**Figure 2:**
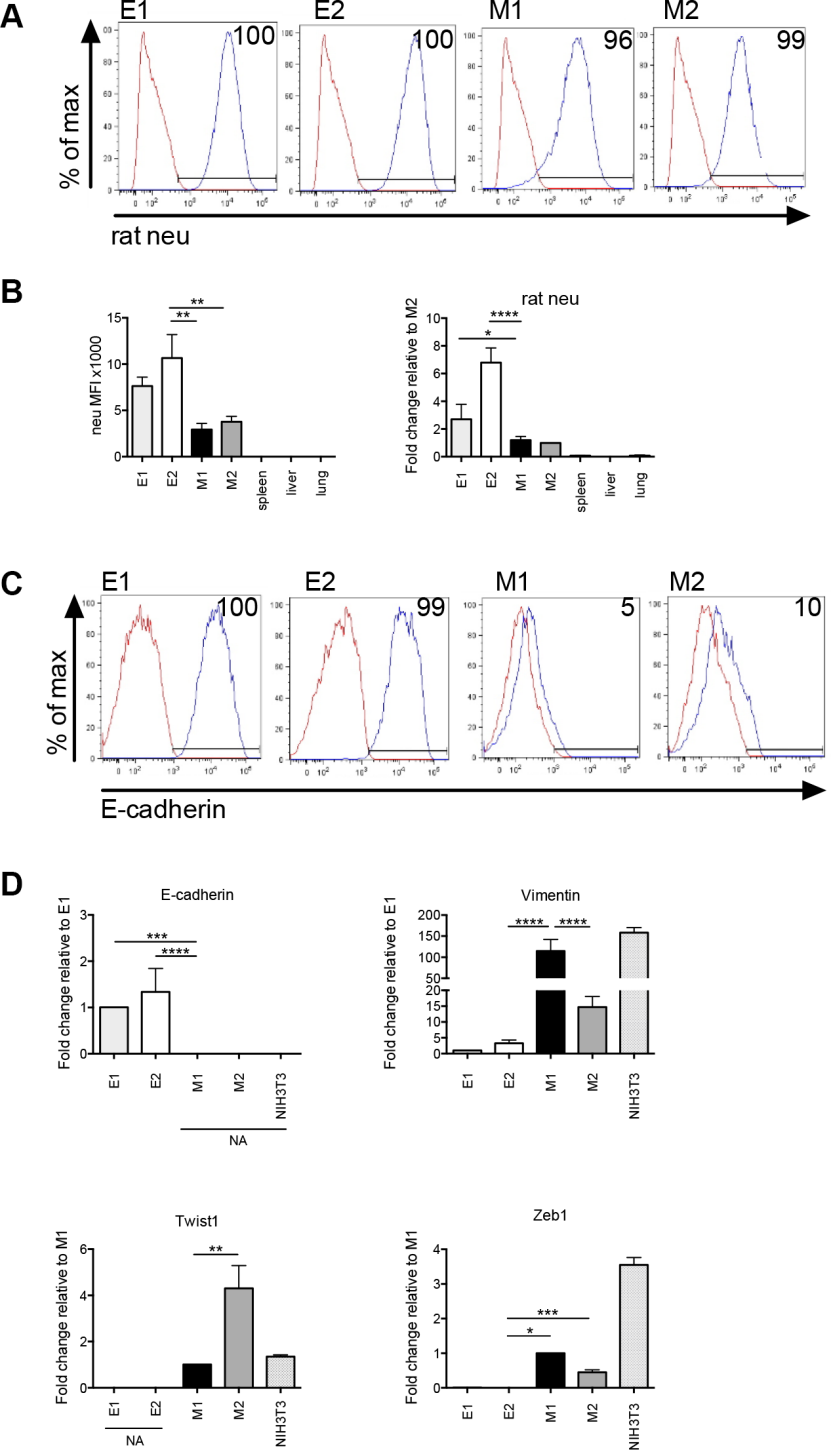
Epithelial and mesenchymal-like clonal cell lines are tumor-derived (**A**) Flow cytometry histograms showing membrane rat neu protein expression. Red histogram = isotype control and blue histogram = neu expression. (**B**) Flow cytometric mean fluorescence intensity (MFI) values of membrane rat neu protein expression (panel 1). The MFI value was obtained by subtracting the MFI of the isotype control from the experimental value. Real-time quantitative PCR (qPCR) of cell line or tissue cDNA using rat neu specific primers (panel 2). For flow cytometry and qPCR, error bars represent repeats of 3 separate experiments. (**C**) Flow cytometric analysis of membrane E-cadherin protein expression. Red histogram = isotype control and blue histogram = E-cadherin expression. (**D**) qPCR showing relative differences in transcript expression of the epithelial marker, E-cadherin, and mesenchymal markers, vimentin, Zeb1 and Twist1 in E1, E2, M1 and M2 cell lines. NIH3T3 cells were analyzed as a mesenchymal cell line control. NA = no amplification signal. Error bars represent the average ΔΔCq from triplicate wells in 3 separate experiments. Statistical analysis was done using ordinary one-way ANOVA with a Tukey multiple comparison test. *p ≤ 0.05, **p ≤ 0.01, ***p ≤ 0.001 and ****p ≤ 0.0001.

To confirm the origin of E and M cells, we stained them with a rat-specific anti-neu antibody and analyzed membrane neu protein expression by flow cytometry. There was 96–100% expression of rat neu (blue histogram as compared to the red isotype control histogram) in the E and M cell lines providing evidence that the cells were tumor-derived (Figure 2A). The mean fluorescence intensity of neu was lower in the M cell lines as compared to the E cell lines, which is consistent with the method by which these cell types were originally separated (Figure 2B, panel 1). Membrane rat neu protein was not expressed on cells harvested from the spleens, livers and lungs of naive Tg/neu mice. We also investigated rat neu transcript expression using cDNA produced from each of the tumor cell lines, as well as lung, liver and spleen cells (harvested from naïve Tg/neu mice). cDNA was amplified by qPCR using a rat-specific neu PCR primer pair, which showed increased expression of rat neu as compared to liver, lung and spleen (Figure 2B, panel 2). These data collectively show that the E and M cells are tumor-derived.

Additional characterization for the E and M cells was performed to determine whether they were epithelial or mesenchymal-like. Molecular analysis of markers that define epithelial (E-cadherin) and mesenchymal cells (vimentin, Zeb1 and Twist1) was conducted. Membrane E-cadherin protein expression was measured by flow cytometry. Greater than 90% of E1 and E2 cells expressed E-cadherin (blue histogram as compared to the red isotype control histogram), whereas less than 10% of M1 or M2 cells expressed membrane E-cadherin (Figure 2C). Transcript expression of epithelial or mesenchymal associated genes in the clonal cell lines was determined for E-cadherin, vimentin, Zeb1 and Twist1. There was an increase in expression of E-cadherin transcript in the E cell lines compared to M cells (Figure 2D). Likewise, vimentin, Zeb1 and Twist1 transcript expression were increased in M cells as compared to E cells. cDNA from NIH3T3 cells was amplified as a mesenchymal cell control. Taken together, these data suggest that the E cells are epithelial and the M cells are mesenchymal-like.

### EMT occurs within 10 days in orthotopically implanted clonal epithelial tumor cells

**Figure 3:**
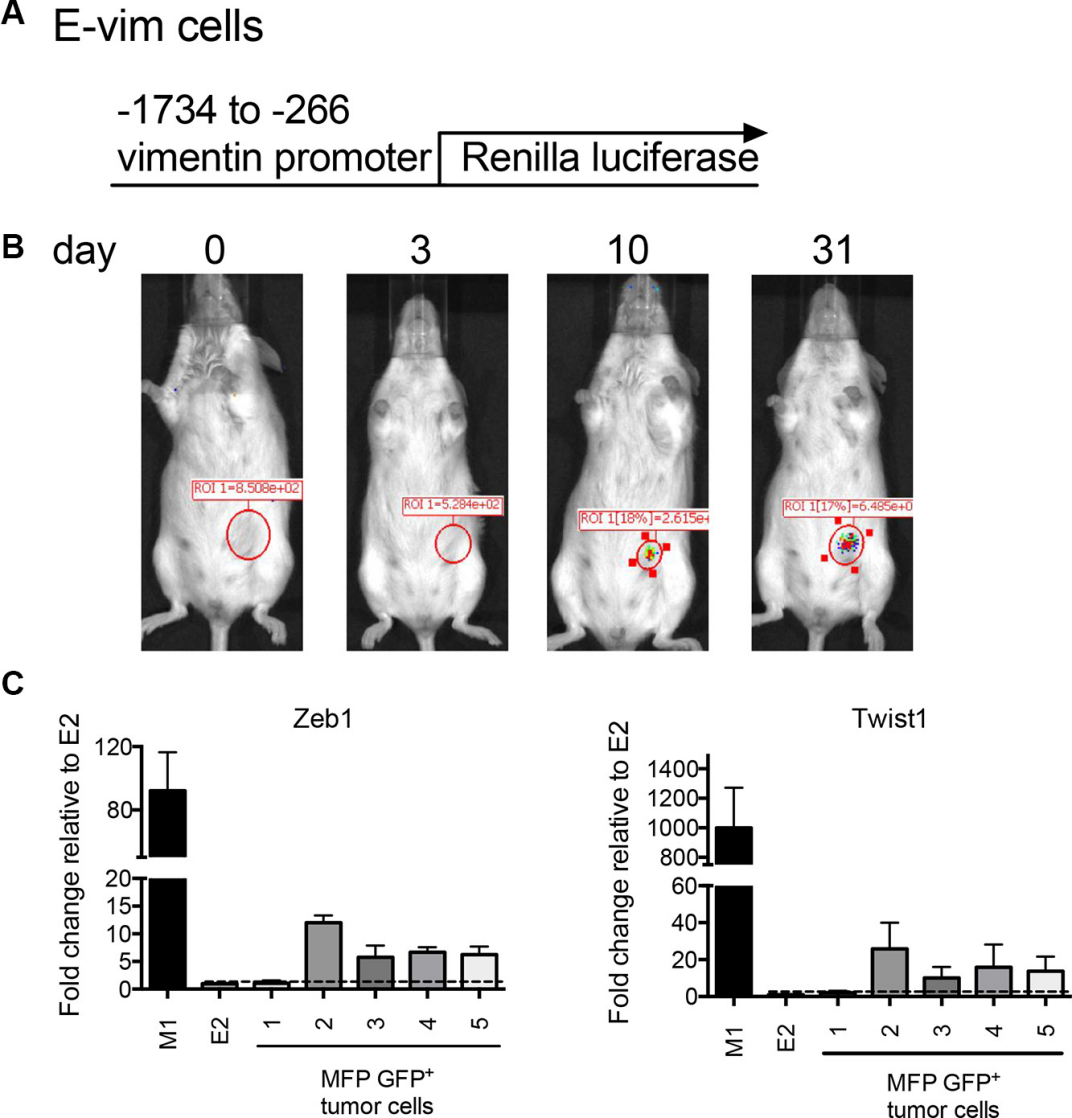
Orthotopically implanted clonal epithelial tumor cells upregulate mesenchymal gene expression over time (**A**) The lentiviral construct used to transduce E2 cells to produce E-vim cells. (**B**) Images show Renilla luciferase bioluminescence on days 0, 3, 10 and 31 following inoculation of 2 × 10^6^ E-vim cells into the MFP. The figure is representative of data obtained from 4 mice. (**C**) Transcript expression of Zeb1 and Twist1 in flow cytometry sorted E2 GFP^+^ tumor cells harvested from the MFP. Five mice were inoculated into the MFP with either 2 × 106 E2 (tumors 2, 3 and 4) or 1 × 10^6^ E2 cells plus 1 × 10^6^ M1 cells (tumors 1 and 5). Tumors were harvested (~200 days after tumor cell inoculation) from moribund mice when tumor size reached 250 mm^2^. Transcript expression is relative to the parental E2 cell line (dotted line).

EMT and MET are two fundamental processes that contribute to tumor progression as reviewed in [32]. We first evaluated EMT events in E cells. To investigate whether E cells undergo mesenchymal changes during tumor growth, E2 cells were transduced with virus expressing Renilla luciferase under the control of the vimentin promoter (referred to as E-vim cells) (Figure 3A). Vimentin is a well-established marker of mesenchymal transition [32]. Therefore, luciferase expression in E-vim cells over time would indicate an EMT transitioning process occurring at the primary tumor site. 2 × 10^6^ E-vim cells were implanted orthotopically into the MFP, and mice were imaged to detect bioluminescence as an indicator of Renilla luciferase activity. Initially, following inoculation of E-vim tumor cells, the biophotonic signal was not detected at the MFP tumor site (Figure 3B). As early as 10 days post tumor inoculation, the bioluminescent Renilla luciferase signal appeared and the signal intensified over time. This data suggests that E-vim cells have turned on vimentin transcription at 10 days implying that they are transitioning to a mesenchymal state *in vivo*.

To investigate whether mesenchymal transcription factors are induced in orthotopically implanted E2 tumor cells, and whether M cells influenced E cell EMT transition, we injected the MFP with either 2 × 10^6^ E2 cells expressing GFP (tumors 2, 3 and 4) or 1 × 10^6^ GFP^+^ E2 cells along with 1 × 10^6^ M1 cells (tumors 1 and 5). MFP tumors from 5 mice were harvested when they reached ~250 mm^2^. GFP^+^ cells were sorted by flow cytometry to recover a purified population of E2 tumor cells. Zeb1 and Twist1 transcript expression in the recovered tumor cells was determined by cDNA qPCR (Figure 3C). In 4 of 5 recovered tumors, there was an increase in Zeb1 and Twist1 transcript in GFP^+^ sorted cells as compared to the parental E2 cell line (Figure 3C dotted line). There was no difference in Zeb1 or Twist1 expression in the recovered E2 tumor cells when mesenchymal-like M1 cells were included in the initial tumor cell inoculum. Along with our *in vivo* analysis, these data collectively suggest that mesenchymal gene transcription induction is an early event in E2 cells at the orthotopic primary tumor site, and already established mesenchymal cells have little effect on this transition process.

**Figure 4:**
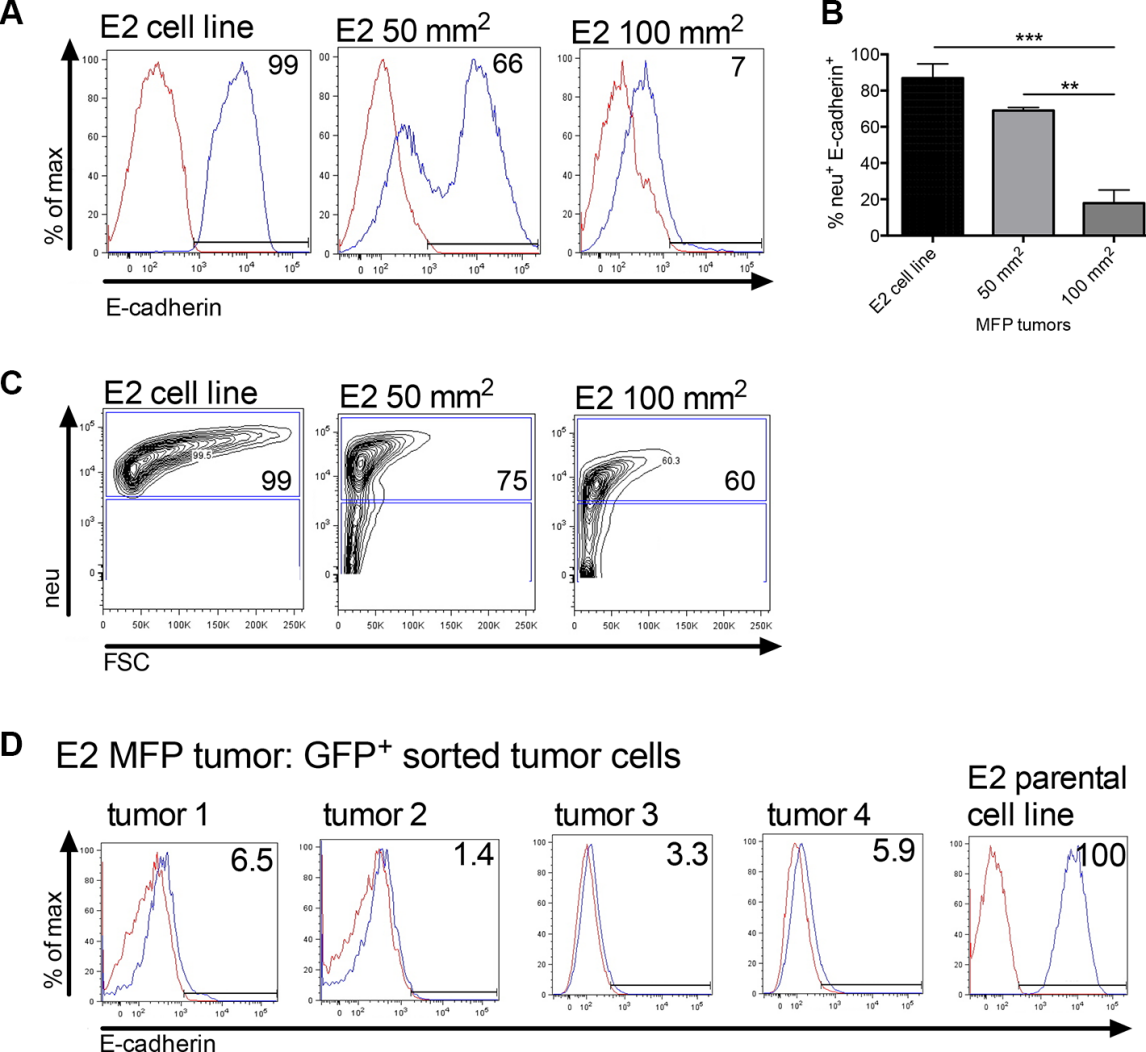
Orthotopically implanted clonal epithelial tumor cells lose E-cadherin expression over time (**A**) A flow cytometric representation of membrane E-cadherin expression in neu^+^ gated E2 tumor cells harvested from MFP tumors at 50 mm^2^ (*n* = 3, day 23) and 100 mm^2^ in size (*n* = 5, day 61). E2 is the parental cell line control. (**B**) Graph of combined flow cytometric data shown in A. Statistical analysis was done using ordinary one-way ANOVA with a Tukey multiple comparison test. **p ≤ 0.01, and ***p ≤ 0.001. (**C**) Flow cytometry showing the neu^+^ cell gate from which cells in A were analyzed. (**D**) Membrane E-cadherin expression on E2 GFP^+^ sorted cells harvested from MFP tumors (~250 mm^2^, day ~200). Mice were inoculated with 2 × 10^6^ GFP^+^ E2 cells (tumors 1 and 2) or a mix of 1 × 10^6^ GFP^+^ E2 and 1 × 10^6^ M1 tumor cells (tumors 3 and 4). MFP = mammary fat pad.

We next investigated loss of E-cadherin expression, a hallmark of EMT in primary tumors. Mice were inoculated with 2 × 10^6^ E2 tumor cells into the MFP and tumors were investigated for membrane E-cadherin expression over time. Tumors harvested at 50 mm^2^ (23 days post tumor inoculation), and 100 mm^2^ (61 days post tumor inoculation) showed progressive decrease in E-cadherin expression (Figures 4A and 4B). E-cadherin expression shown in Figure 4A was analyzed from neu^+^ gated tumor cells (as shown in Figure 4C) to ensure tumor cell specificity. Large MFP tumors at 250 mm^2^ (2 × 10^6^ GFP^+^ E2 cells or 1 × 10^6^ GFP^+^ E2 cells mixed with 1 × 10^6^ non-tumorigenic M1 cells) harvested from moribund mice were also analyzed for E-cadherin expression. The membrane E-cadherin expression was analyzed on flow cytometry sorted GFP^+^ E2 cells (Figure 4D). Regardless of whether only E2 cells were inoculated (tumors 1 and 2) or E2 cells were combined with M1 cells (tumors 3 and 4) the E2 GFP^+^ cells recovered from the orthotopic tumor had similarly reduced membrane E-cadherin protein expression when compared to the parental E2 cell line. Collectively, these data suggest that clonal epithelial tumor cells growing at the orthotopic site are undergoing transition into a mesenchymal state irrespective of their proximity to established mesenchymal cells.

### E2 cells metastasize and membrane E-cadherin is re-expressed at the metastatic site

**Figure 5:**
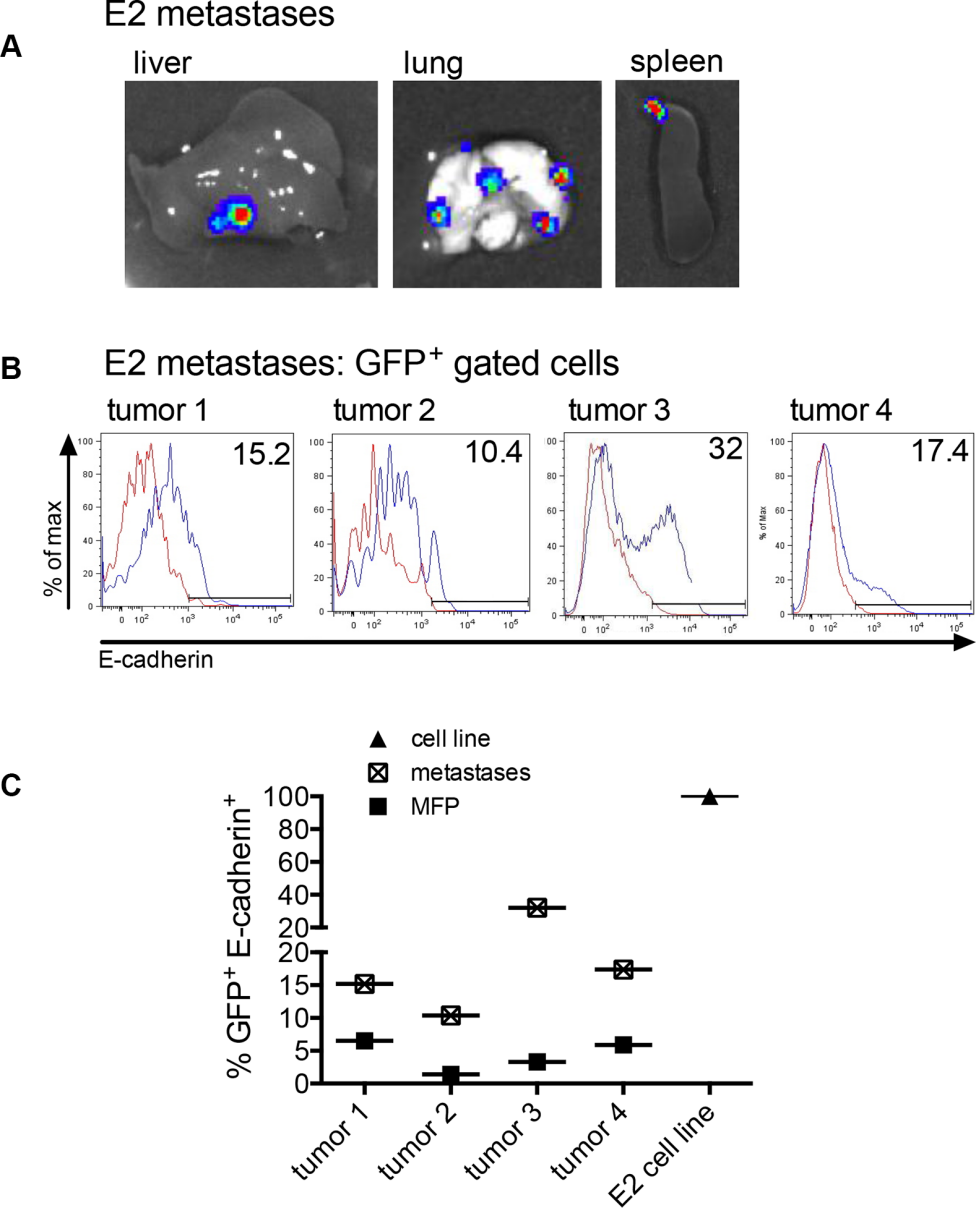
Metastasized clonal epithelial tumor cells re-express E-cadherin (**A**) A representation of firefly luciferase bioluminescent signal in organs of mice injected into the MFP with 2 × 10^6^ GFP^+^ E2 cells expressing firefly luciferase or 1 × 10^6^ GFP^+^ E2 cells expressing firefly luciferase mixed with 1 × 10^6^ M1 cells. (**B**) Membrane E-cadherin expression on GFP^+^ flow cytometry-gated cells recovered from metastatic organs. (**C**) The graph showing the difference in E-cadherin expression in E2 cells harvested from the MFP (shown in Figure 4D) and E2 cells harvested from the corresponding metastasis (Figure 5B). E2 is the parental cell line control.

We next investigated whether the E2 cells that underwent metastasis showed changes in E-cadherin expression at the metastatic site. Mice injected with 2 × 10^6^ E2 or 1 × 10^6^ E2 mixed with 1 × 10^6^ M1 cells into the MFP (same mice as in Figure 4) were analyzed for metastasis of E2 cells. In addition to the primary tumors, the spleens, livers and lungs were harvested and imaged for bioluminescence. The bioluminescent signal in Figure 5A is a representation of firefly luciferase activity detected from E2 cell metastases in the liver, lung and spleen. For mice inoculated with E2 cells, organ metastasis was detected in 13 of 16 mice. For mice that received E2 cells mixed with M1 cells, organ metastasis was detected in 12 of 15 mice. These data show no impact on E2 metastasis when epithelial tumor cells were mixed with non-tumorigenic mesenchymal cells in the initial tumor cell inoculum. E-cadherin protein expression was analyzed by flow cytometry on GFP^+^ gated tumor cells derived from the organ metastases (Figure 5B). The E-cadherin membrane expression was higher in the E2 cells recovered from the metastases as compared to the E2 cells harvested from the corresponding MFP primary tumor (Figure 5C). Since the tumor cells were clonal, the loss of E-cadherin expression in cells in the MFP and a gain in E-cadherin expression at the corresponding metastases shows direct *in vivo* evidence of EMT and MET.

### E-cadherin expression in M1 cells does not rescue tumor formation at the orthotopic site

**Figure 6:**
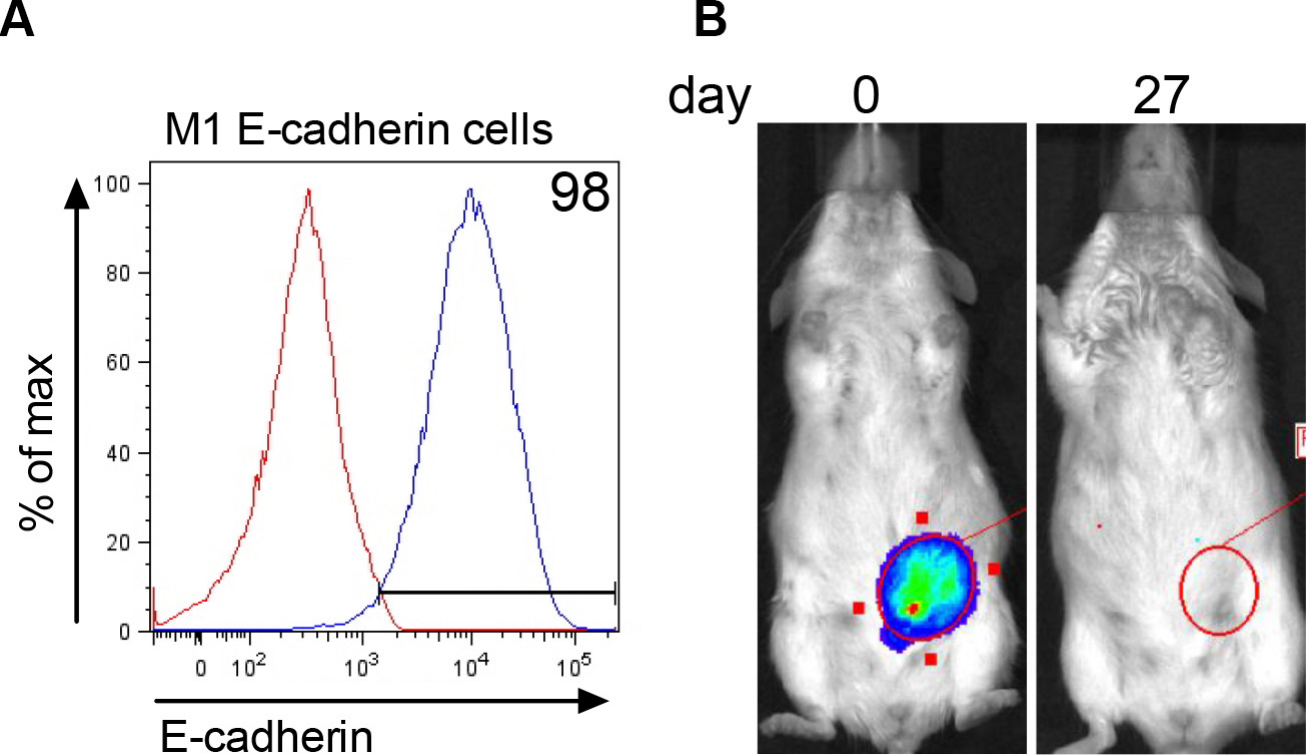
E-cadherin expression in M1 cells does not rescue tumorigenesis (**A**) Stable expression of E-cadherin in M1 cells transfected with Addgene plasmid #18804 expressing murine E-cadherin. Red line = E-cadherin expression in parental M1 cell line and blue line = membrane E-cadherin expression in transfected M1 cells. (**B**) Renilla luciferase bioluminescent signal in the MFP immediately (day 0) after injection of 2 × 10^6^ M1 cells expressing E-cadherin and 27 days later. The figure represents data obtained from 9 mice.

Since E-cadherin was re-expressed in colonized metastatic tumor cells, we were interested to test whether E-cadherin expression in M1 cells would rescue the ability of these cells to form tumor. M1 cells stably expressing E-cadherin (Figure 6A) and Renilla luciferase were inoculated into the mammary fat pad and followed for tumor progression using biophotonic imaging. Tumor cells were present in the mammary fat pad immediately after inoculation (Figure 6B, day 0). By day 27, the luciferase signal disappeared. These data show that E-cadherin expressing M1 cells do not persist to form tumor in the mammary fat pad.

## DISCUSSION

To our knowledge, these data are the first to show *in vivo* evidence of both EMT and MET by tracking and analyzing clonal tumor cells in the primary orthotopic tumor site and the corresponding metastatic site. In this study, we show EMT changes, including loss of E-cadherin membrane expression, in clonal epithelial breast tumor cells growing at the primary orthotopic site. *In vivo* evidence of MET is shown by re-expression of E-cadherin in metastasized cells.

The ability to clone two epithelial and two mesenchymal-like cell lines from the same spontaneous tumor speaks to the heterogeneity of the tumor cell populations, a fact that is increasingly appreciated in the literature [32, 33]. E-cadherin has been implicated as a regulator of tumor cell colonization [9, 13, 14, 31, 32, 34–41]. In prostate cancer, knockdown of E-cadherin in PC-3/Mc cells reduced their ability to form spheroids and colonize in the lung [31]. Likewise, overexpression of E-cadherin in mesenchymal-like prostate cancer PC-3/S cells rescued the ability of these cells to form spheroids. However, whether E-cadherin expression is required for tumor cell colonization remains to be determined. In our study one of the mesenchymal cell lines (M2) colonizes to form tumor, while the other mesenchymal cell line (M1) does not. Both cell lines have low E-cadherin expression. In our model, stable expression of E-cadherin in the non-tumorigenic M1 cells did not rescue the ability to colonize tumor (Figures 6A and 6B). Interestingly, human xenografted cancer cells that lacked E-cadherin were also able to colonize to form metastastic lesions [23]. These data suggest that factors other than E-cadherin are required for tumor cell colonization. It is possible the E-cadherin expression is not causal, but a marker of events. Other causal candidate genes that may play a role in tumor cell colonization in low E-cadherin expressing cells will need to be identified and tested.

It is possible that M1 cells have a transcriptome that does not allow for tumor cell colonization and growth. Support for this hypothesis comes from the microarray data shown in Figure 1D. The heat map provides information that correlates gene expression with differences in tumorigenic potential. The transcripts in the heat map fulfill two criteria. There is at least a 10-fold difference in expression between the two mesenchymal-like cell lines (M1 and M2), and the transcripts are reciprocally regulated in cells that do and do not colonize to form tumor. It is intriguing that transcript expression is similar in all the tumorigenic cell lines regardless whether they are epithelial (with high E-cadherin expression) or mesenchymal-like (with low E-cadherin expression). While any of the genes shown in Figure 1D may play a role in tumor cell growth, there are several that are of particular interest. Decorin, follistatin and insulin-like growth factor binding protein 7 are proteins with upregulated expression in the non-tumorigenic cell line as compared to the tumorigenic cell lines. Decorin (Dcn) is an extracelluar matrix proteoglycan regarded as a tumor suppressor through its role in inhibiting tumor development as reviewed in [42]. Decorin inhibits molecules that enhance cancer growth and progression including TGF-β, receptor tyrosine kinases, platelet-derived growth factor, lipoprotein receptor-related protein-1 (LRP-1, a connective tissue growth factor), thrombospondin and Wnt-1-induced secreted protein 1. Follistatin (Fst) is an extracellular glycoprotein that negatively regulates activin (a TGF-β family member) and suppresses cell proliferation [43]. Follistatin has been show to suppress tumor growth by inducing cancer cell apoptosis [44]. Expression of insulin-like growth factor binding protein 7 (Igfbp-7) in melanoma cells was shown to inhibit cell growth due to apoptosis [45]. Equally intriguing is the upregulated expression of angiopoietin-like protein-1 (Angptl1) and angiopoietin-like protein-2 (Angptl2) in non-tumorigenic and tumorigenic cells, respectively. Angptl1 (also referred to as angioarrestin) has been shown to reduce cell adhesion [46] and inhibit tumor cell growth *in vivo* [47, 48]. Tumors that are derived from cells genetically induced to overexpress Angptl1 are smaller than those produced by control cells. Angptl2 is a mediator chronic inflammation, increased oxidative stress and tumor progression [49–51]. Angptl2 may promote tumorigenesis through reactive oxygen induced inactivation of tumor suppressor phosphatases (PTEN and shp2) [52] as well as p53 and IKB-α tumor suppressor genes [49]. Understanding the mechanistic role(s) that these genes (and others listed in Figure 1D) have in promoting or inhibiting tumor growth is paramount to the understanding of tumor cell colonization and metastasis.

It is possible that epithelial carcinoma tumor cells with high metastatic potential respond to environmental cues resulting in transient rounds of EMT and MET [32]. This cellular plasticity would allow for both motility and colonization depending on the state of differentiation. It is known that EMT can be induced in tumor cell lines by external factors. Some of these include transforming growth factor beta (TGF-β), [1], hepatocyte growth factor (HGF) [2], epidermal growth factors (EGF) [3], insulin-like growth factors (IGF) [4], and fibroblastic growth factor (FGF) [5, 7]. There is also evidence that mesenchymal cell-derived factors induce EMT. Cooperation between mesenchymal-like tumor cells and epithelial tumor cells facilitated local invasiveness of epithelial tumor cells. When non-invasive PC-3/Mc prostate cancer cells were co-cultured with invasive mesenchymal-like PC-3/S cells or NIH3T3 fibroblasts, the PC-3/Mc cells became invasive [31]. In the current study we tested whether non-tumorigenic mesenchymal cells impacted EMT and metastasis of E2 cells by adding M1 cells to the E2 orthotopic tumor inoculum. There were no detectable differences in EMT in the E2 orthotopic tumors or the incidence of metastasis. It is possible there was no influence because M1 cells did not persist *in vivo* as they were not detected in the MFP as early as 10 days following tumor cell inoculation (Figure 1C). Identifying the cells and/or factors in the tumor microenvironment that facilitate EMT and MET in this model system is an important area of future study.

In summary, by tracking changes in E-cadherin expression using a unique system of clonal tumor cells our data shows direct *in vivo* evidence of mesenchymal transitioning (EMT) occurring in the primary orthotopic site for epithelial cells, and lack of influence of mesenchymal cells to this process. We also show re-expression of E-cadherin in epithelial cells (MET) at the distant metastatic site. Finally, in our model, E-cadherin expression in the non-tumorigenic M1 cells was not sufficient to restore tumor cell colonization. Collectively, our data shows a dynamic plasticity of EMT and MET in murine breast carcinoma tumor cells. Understanding the mechanisms that influence the differentiation state of carcinoma tumor cells is a goal for future research.

## MATERIALS AND METHODS

### Mice

Female FVB/N Tg (MMTV/neu) 202MUL/J (referred to as Tg/neu) mice were purchased from the Jackson Laboratory (Bar Harbor, ME) and housed in the Biomedical Resource Center at the Medical College of Wisconsin. Primary clonal syngeneic murine mammary tumor cells (5 × 10^4^ or 2 × 10^6^) suspended in phosphate buffered saline (PBS) were injected into the “D” mammary fat pad (MFP) to produce orthotopic mammary tumors and subsequent metastases. Tumor cells (2 × 10^6^) suspended in PBS were also injected via the tail vein to determine the ability of tumor cells to colonize the lung. Routine caliper measurements and biophotonic imaging were used to determine tumor growth. When tumors exceeded 250 mm^2^, mice were considered moribund and euthanized. All experiments were approved by the Medical College of Wisconsin Institutional Animal Care and Use Committee (IACUC).

### Mouse tumor primary cell lines

To produce primary mammary tumor cell lines, a spontaneous mammary tumor was harvested from a female Tg/neu mouse. The tumor was processed to generate a single cell suspension by compression through a 1.0 mm pore mesh screen. To subfractionate the isolated mammary epithelial tumor cells into epithelial and mesenchymal compartments, cells were stained with a fluorescein isothiocyanate (FITC)-conjugated CD24 specific monoclonal antibody (clone M1/69, eBioscience, San Diego, CA), a mouse anti-c-ErbB2/c-neu monoclonal antibody (Clone 7.16.4, Calbiochem-EMD Chemicals, Gibbstown, NJ) plus a phycoerythrin (PE)-conjugated goat anti-mouse secondary antibody (Jackson ImmunoResearch, West Grove, PA), and then sorted by flow cytometry based on neu and CD24 expression using a FACSAria III flow cytometer and FACSDiva software (Becton-Dickinson, Franklin Lakes, NJ). Sorted epithelial tumor cells with high CD24 and high neu expression were cultured in RPMI supplemented with 20% fetal bovine serum (FBS), 12 mM HEPES buffer, 2 mM L-glutamine, 10 μM non-essential amino acids, 1 mM sodium pyruvate, 100 μg/ml streptomycin and 100 U/ml penicillin. Mesenchymal-like tumor cells with low CD24 and low neu expression were cultured in MEGM^®^ (Lonza, Switzerland) supplemented with 2% FBS and proprietary concentrations of bovine pituitary extract, epidermal growth factor, insulin, hydrocortisone, gentamycin, and amphotericin [7]. Cells were cultured by limiting dilution to produce two epithelial (E1 and E2) and two mesenchymal-like clonal cell lines (M1 and M2). To produce a clonal epithelial cell line expressing firefly luciferase and emerald green fluorescent protein (GFP), the E2 cell line was transfected by nucleofection with pcDNA3.1/Hygro^−^ (Thermo Fisher Scientific,) encoding firefly luciferase and transduced with virus derived from pLenti6.4/Promoter/MSGW/EmGFP vector (Thermo Fisher Scientific). The E2 cell line expressing firefly luciferase and GFP was selected by culturing the cells in 200 μg/ml hygromycin and 3 μg/ml blasticidin. To produce mesenchymal-like cell lines expressing Renilla luciferase and mCherry, clonal M cells were transduced with virus derived from pLVX-mCherry-N1 (Clontech) encoding Renilla luciferase. Transduced cells were selected by culture in 2 μg/ml puromycin. To produce an epithelial cell line encoding Renilla luciferase under the control of the murine vimentin promoter (E-vim), the lentiviral vector cytomegalovirus (CMV) promoter region of the Renilla luciferase pLVX-mCherry-N1 vector was replaced with nucleotides −271 to −1518 of the mouse vimentin promoter region. The CMV promoter region was removed by restriction digest with ClaI and XhoI. The mouse vimentin promoter was amplified from M1 cDNA using primers: forward 5′ CCTCCTATCGATAATGGACCCATCTCCCAGTTTGT 3′ and reverse 5′ CCTCCTGTCGACCAGTGCG CTGCCCAGAC 3′. The PCR product was digested with ClaI and XhoI and ligated to the ClaI, XhoI digested vector. Clonal E2 cells were transduced with virus produced from this vector and selected in culture with 2 μg/ml puromycin. To express E-cadherin in the non-tumorigenic M1 cell line, cells were transfected with the Addgene pWZL Blast mouse E-cadherin (Plasmid #18804) [14]. Transfected cells expressing E-cadherin were selected in 3 μg/ml blasticidin.

### Microscopy

For bright field microscopy, cells were imaged using a 10× objective (Zeiss) on a Zeiss Axiovert 200 M microscope. Images were analyzed using AxioVision rel 4.8 (Zeiss) software.

### Flow cytometry

Neu expression was determined by staining clonal tumor cell lines with a rat-specific mouse anti-c-ErbB2/c-neu monoclonal antibody (Clone 7.16.4, Calbiochem-EMD Chemicals, Gibbstown, NJ) and a phycoerythrin (PE)-conjugated goat anti-mouse secondary antibody (Jackson ImmunoResearch, West Grove, PA). For neu expression, the PE-conjugated mouse IgG2aκ clone eBM2a was used as an isotype control. Membrane E-cadherin was detected using an eFluor-660 conjugated anti-CD324 antibody (clone DECMA-1; e-Bioscience) and the eFluor-660 conjugated rat IgG1 (clone eBRG1; eBioscience) was used as an isotype control. To obtain a purified population of GFP^+^ positive tumor cells, GFP expressing E2 cells were harvested and processed into single cell suspensions using the Tumor Dissociation Kit and the gentle MACS Dissociator (Miltenyi Biotec) per the manufacturer's protocol. GFP positive cells were sorted from GFP negative cells using a FACSAria III flow cytometer with FACSDiva software (Becton-Dickinson). All antibody-stained and fluorescent cells were detected on a LSR II flow cytometer (BD Biosciences) and analyzed using FlowJo software (Treestar, Inc.).

### Real time PCR (qPCR)

For qPCR of cDNA: Tumor cells were suspended in Trizol^®^ and immediately frozen at −80°C. RNA was processed according to the Trizol^®^ reagent manufacturer's protocol (Thermofisher). The RNA optical density and ratio of absorbance at 260 and 280 nm was determined using a Nanodrop-1000 spectrophotometer (Thermo Fisher Scientific). cDNA was synthesized with 2 μg of RNA using the Quantitect reverse transcription cDNA kit (Qiagen) per manufacturer's protocol. cDNA at a 1:10 dilution was amplified in a 20 μl reaction volume using a CFX C1000 real-time thermal cycler (Bio-Rad) with the SsoFast™ Eva Green^®^ Syber Green Supermix (Bio-Rad) under the following amplification protocol: 95°C for 30 seconds followed by 39 cycles of 95°C for 3 seconds and 60°C for 3 seconds. Samples were then heated to 95°C for 10 seconds followed by a melt curve analysis from 65°C to 95°C with measurements in 0.5 degree increments every 5 seconds. The 18 S reference gene was amplified using QuantiTect 18 S primers (Qiagen). All reactions were done in triplicate wells with duplicate no template control wells. To calculate fold-change in gene expression, the average ΔΔCq values from triplicate wells were combined from at least 3 experiments. The primer sets used are as follows (listed 5′ to 3′). Rat neu specific: forward GGCATTGCTCCGCTGAGG reverse CACTGAGGTCACGGAGACTGT; Zeb1: forward GCAGGAGCCGCCAGTGAAGG reverse TGGGTGG CGTGGAGTCAGAGT; Twist1: forward GGAGCTCCC CACCCCCTCTG reverse TCCACGGGCCTGTCTC GCTT; vimentin: forward CGCCAGGCCAAGCAGGA GTC reverse CTCATCCTGCAGGCGGCCAA; E-cadherin: forward GCCGGAGAGGCACCTGGAGA reverse GCGCGGACGAGGAAACTGGT. Primer efficiency/r^2^ values were as follows: neu 107.6%/0.990; Zeb1 111.6%/0.998; Twist1 97.2%/0.915; vimentin 99.0%/0.995 [7] and E-cadherin 92.6%/0.967. Relative quantity values from triplicate wells were combined from at least 3 separate experiments.

### Biophotonic imaging

Tumors produced from firefly and Renilla luciferase-expressing cells were imaged for bioluminescence using a Xenogen IVIS imaging system. To detect Renilla luciferase bioluminescence, mice were injected intravenously with 15 μg coelenterazine (Nanolight) diluted in 300 μl PBS. To detect firefly luciferase bioluminescence, mice received 1 mg D-luciferin (Regis Technologies) dissolved in 200 μl PBS by intraperitoneal injection (i.p.). Regions of interest (ROI) surrounding the tumor bioluminescent signal were manually drawn using LIVING IMAGE software Version 2.6 (Xenogen). Results are reported as ROI total photon flux.

### Affymetrix RNA microarray

RNA from each cell line (E1, E2, M1 and M2) was prepared using the RNeasy^®^ Micro Kit (Qiagen). The purified RNA was quantified with a Nanodrop 1000 UV/Vis-spectrophotometer (Thermo Scientific, Wilmington, DE). Equal concentrations of RNA from each cell line were sent to the Genomics Core Facility at Brown University (Providence RI) and analyzed on Affymetrix^®^ Mouse MoGene-1_0-st-v1 arrays. Microarray data were exported from Affymetrix GCOS software as CEL files. The data has been deposited in NCBI's Gene Expression Omnibus and are accessible through GEO Series accession number GSE81033. (https://www.ncbi.nlm.nih.gov/geo/query/acc.cgi?acc=GSE81033).

### Statistical analysis

Data were compared using ordinary one-way ANOVA non-parametric analysis with the Tukey multiple comparison test. Analyses were calculated with Prism graph pad 6.0 software (GraphPad, San Diego, CA).
